# The Effects of Temperament on Depression According to the Schema Model: A Scoping Review

**DOI:** 10.3390/ijerph15061231

**Published:** 2018-06-11

**Authors:** Charmaine Ruling Lim, Joanna Barlas, Roger Chun Man Ho

**Affiliations:** 1 Department of Psychology, James Cook University Singapore, 149 Sims Drive, Singapore 387380, Singapore; joanna.barlas@jcu.edu.au; 2 Department of Psychological Medicine, National University Hospital, Level 9, NUHS Tower Block, 1E Kent Ridge Road, Singapore 119228, Singapore; pcmrhcm@nus.edu.sg

**Keywords:** temperament, personality, major depressive disorder, depression, schema therapy, schema model, scoping review

## Abstract

*Background*: Recent studies have shown that not every depressed patient responds to Cognitive Behavioral Therapy, and some of those who do relapse upon termination. Due to its dual focus on the past and present, Schema Model (SM) represents a promising alternative model to understand depression. However, studies examining SM often operationalize the same construct differently, resulting in inconsistent evidence of change. There is no known review clarifying (1) how best to assess schema constructs; and (2) the relevant pathways to depression, without which, the empirical basis for SM cannot be examined. *Methods*: A scoping review was conducted in accordance to PRISMA guidelines to map evidence of the relationship between constructs of SM and depression, and measures used to assess the constructs. 2463 articles were identified with 49 primary research studies included. This paper is a subset of the scoping review and focuses on the five studies examining effects of temperament on depression. *Results*: Two models were used to operationalize temperament: The Five Factor Model (FFM) and the Psychobiological Model of Personality (PBM). The variables of neuroticism and harm avoidance were positively associated with depressive symptoms while self-directedness and cooperativeness were negative associated with depressive symptoms. *Conclusion*: The FFM is more suited to operationalize temperament in studies of SM and depression due to its theoretical compatibility with SM, established psychometric properties of its measures, and widespread use among studies of SM. Out of the five factors in the FFM, only neuroticism exerts direct and indirect effects on depression. These findings are limited by homogeneous sampling, hence future research studies should consider extending it to adult clinical samples. Nevertheless, this review represents a first step in the systematic examination of the empirical basis of SM and a contribution to treatment innovation and practice for depression.

## 1. Introduction

### 1.1. About Depression

Depression is one of the most prevalent and debilitating mental disorders. It is an important public health issue, as 322 million people of all ages worldwide suffered from depression in year 2017 [1,2]. Depression causes suffering not only for individuals, but also for their families and society—in year 2010, the estimated economic costs of depression (e.g., workplace, suicide-related costs) stood at $210.5 billion [3]. While depression is commonly treated with a combination of anti-depressants and psychotherapies [2], the former is associated with adverse events such as side effects, discontinuation symptoms, and potential interactions with concomitant medications or physical health problems [2]. Hence, there is a need to improve understanding of psychological treatments for depression.

### 1.2. Psychological Treatments for Depression

Cognitive Behavioral Therapy (CBT) is the prevailing form of psychotherapy to treat depression due to its strong evidence base [4,5]. However, findings from the National Institute of Mental Health Treatment of Depression Collaborative Research Program revealed that only 39% of treated individuals had recovered from depression [6]. When followed up over an 18-month period, only 24% of treated individuals remained well [6]. Additionally, the effects of CBT might be overestimated in current literature. A recent meta-analytic study reported strong and highly significant indications of publication bias among studies examining CBT and other psychological treatments for depression: After its authors adjusted for publication bias, the overall effect size of CBT reduced from 0.69 to 0.49 [7].

Given this, some have argued that the effectiveness of CBT (for depression) had been overstated in the literature [8]. The growing awareness of CBT’s limitations in treating depression has led some authors to re-examine gaps in Beck’s Cognitive Model of Depression [6,9]. Major criticisms of Beck’s model include its limited view of emotions, inadequate consideration of interpersonal factors, under-emphasis on therapeutic alliance, and over-emphasis on clients’ ability to engage in conscious and controlled processing [10]. While personal history and family history of depression have been elucidated as important factors that significantly impact the individual’s course of depression and response to treatment [2], Beck’s conceptualization of depression may not have adequately addressed these factors, leading to an under-emphasis in CBT on addressing underlying dysfunctional schemas [11]. Accordingly, the persistence and relapse of depressive episodes have been attributed to the activation of unaddressed underlying dysfunctional schemas [11].

In conclusion, Beck’s Cognitive Model of Depression [9] may not adequately explain depression. Consequently, one of the most important challenges facing research and treatment in depression lies in understanding factors linked to relapse, with the goal of discovering more effective means to lower recurrence of depressive episodes [12].

### 1.3. New Therapeutic Approaches to Depression

A strong psychological theory of depression should make a significant contribution to treatment innovation and practice [12]. A third wave of cognitive therapies, such as Mindfulness Based Therapies, Dialectical Behavioral Therapy, Acceptance Commitment Therapy, and Schema Therapy (ST), has been developed to address various limitations of CBT. In contrast to other third-wave cognitive therapies which focus on the present, ST is differentiated by its dual-focus on both past and present in the development of psychopathology. Hence, ST was deemed to be most appropriate in addressing limitations of CBT in treating depression.

### 1.4. About the Schema Model

Schema Model (SM) [13,14] is the underlying theory of ST and expands on Beck’s Cognitive Model with ideas from psychodynamic theory, particularly object relations and attachment theories [15,16]. As SM conceptualizes clients’ psychological problems as being maintained by complex characterological underpinnings, ST correspondingly places a greater emphasis on identifying and treating these developmental origins [11].

**Figure 1:**
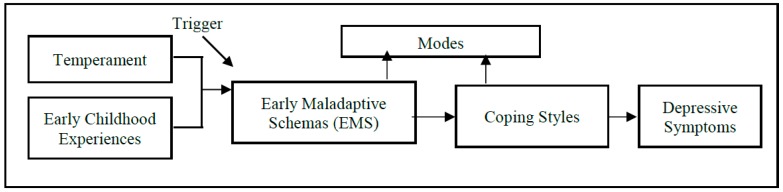
Pictorial Representation of the Schema Model [14]. This has been simplified to exclude other potential direct and indirect relationships between variables.

SM postulates that each child has core emotional needs. Bi-directional interactions between the child’s early experiences and his/her temperamental disposition may result in these needs not being met. When the child’s core emotional needs are not met, he/she is at risk of developing Early Maladaptive Schemas (EMS). EMS represent fundamental, unconditional assumptions that an individual has about themselves and their relationships with significant others. As EMS often represent threats to a child, they learn to cope through both adaptive and maladaptive behaviors (coping styles), the latter leading to and maintaining psychopathology in adulthood [14,17,18]. Individuals also cope with EMS through modes, which are moment-to-moment emotional states and coping experiences of an individual [14]. The concept of modes was primarily developed to explain the continual shift from one extreme affective state or coping response to another observed among clients with Borderline Personality Disorder [14]. See Figure 1 for a pictorial representation of SM.

Correspondingly, ST focuses on “healing” EMS and breaking patterns of maladaptive coping styles for clients to meet their emotional needs in healthier ways [14]. As SM postulates that EMS present in a continuum, ST can be flexibly applied in a brief, moderate or intensive fashion to address different levels of needs and symptom severity [14]. ST normalizes rather than pathologies psychological disorders—everyone has schemas, coping styles, and modes—they are just more extreme and rigid in patients [14].

While many studies have examined the efficacy of ST [11,19,20], far fewer studies have examined the empirical basis of its conceptual model. At present, while parts of the SM have been partially supported by various studies, no study has examined it in its entirety. A key barrier to doing so lies inherent within existing research studies, specifically different operationalization of the same construct, resulting in inconsistent evidence of change [19]. Hence, before SM and its pathways can be empirically tested, measures of the individual constructs must be first assessed for its psychometric properties and theoretical alignment with SM.

A literature review is required to address this research gap. Scoping reviews are a form of systematic literature review that specifically examines a broad area to identify gaps in research knowledge base [21], clarify key concepts [22], or report on types of evidence that address and inform practice in the field [23]. Specifically, scoping reviews can be used to clarify working definitions, map available evidence, and to collate a list of types and details of measures used to assess a given construct from an evidence base [24]. 

Consequently, a scoping review is most appropriate to address the above research gap. Hence, a scoping review was conducted on existing research on the developmental origins and maintaining pathways of depression as explained by the SM. Its findings will generate separate streams of synthesis, each of which addressing a different construct with differing types of evidence. Therefore, one (or two related) constructs of the SM will be examined in depth in separate articles, leading to three distinct articles. This article (i.e., Part 1) discusses the role of temperament in SM’s explanation of depression. Specifically, this paper covers findings of the scoping review of the relationship between temperament and depression as according to the SM, a description of the two main models in this area, and assesses suitability of each model to measure temperament as conceptualized in SM.

The objectives, inclusion criteria and methods for this scoping review were specified in advance and documented in the Joanna Briggs Institute approach to conducting and reporting scoping reviews protocol [24]. This protocol is congruent with the Preferred Reporting Items for Systematic Reviews and Meta-Analyses (PRISMA-ScR) checklist.

## 2. Scoping Review

### 2.1. Objectives and Questions

#### 2.1.1. Objectives 

The primary objective of this scoping review is to provide an overview of existing research on the developmental origins and maintaining pathways of depression as explained by SM. Its secondary objective is to map all instruments used by the above primary research studies to measure constructs of SM.

#### 2.1.2. Questions 

This is extrapolated to the following two research questions: (1) What are the supported and unsupported pathways for depression according to SM? (2) What instruments have been used by these primary research studies to measure constructs of SM?

### 2.2. Inclusion and Exclusion Criteria

#### 2.2.1. Types of Participants 

Only studies published in English were considered for inclusion in the scoping review as there were no resources to translate articles published in languages other than English. Participants of all ages were included in the scoping review. Participants with any degree of depressive symptoms (i.e., negative affect, undiagnosed depression, depression as primary diagnosis, depression as secondary diagnosis) were included as long as the study had explicitly measured depressive symptoms. Participants with co-morbid conditions were included as long as depressive scores were separately reported. For example, a study that reported depression scores on the Depression Anxiety Stress Scale-21 (DASS-21) will be included, whereas a study that included only combined scores on the DASS-21 will be excluded.

#### 2.2.2. Concept 

Studies included in the scoping review must examine developmental and maintenance pathways to depression within a schema framework (i.e., variables must be schema constructs such as “temperament”, “toxic/early childhood experiences”, “early maladaptive schemas”, “coping styles”, “modes”, and “stressors”). These constructs can be measured by any instrument.

#### 2.2.3. Context 

The context of the scoping review was set broadly, i.e., across any country and setting including acute care, primary care and community. This is as schema constructs were theorized as presenting in a continuum [14].

#### 2.2.4. Outcome 

The first desired outcome of the scoping review is to provide an overview of the SM for depression based on the current evidence base. Specifically, the scoping review would list all associated and ruled out variables for each construct. The second desired outcome is to provide a comprehensive listing of all measures used by researchers to measure constructs of SM for depression.

#### 2.2.5. Types of Sources

Only primary research studies, including dissertations, were included in this scoping review. Secondary sources such as books, book chapters, opinions, commentaries, editor’s comment, were excluded from this scoping review.

### 2.3. Search Strategy

#### 2.3.1. Pre-Identification Stage

An initial search of two databases relevant to the topic was conducted. The electronic bibliographical databases of “PsycINFO” and “MedLine” were selected based on their relevance to psychotherapy studies. Keywords of “Schema + Model” and “Schema + Therapy” were used. An analysis of text words contained in the title and abstract of retrieved papers was conducted before the following final search terms were determined: “Schema + Therapy” OR “Schema + Model” OR “Schema + Focused + Therapy” OR “Schema + Mode + Model” OR “Early + Maladaptive + Schemas” OR “Young + Schema + Questionnaire” OR “Schema + Mode” OR “Schema + Mode + Inventory”. The pre-identification stage was conducted by the first author (CL).

#### 2.3.2. Identification Stage

A second full search using the above keywords was undertaken across six electronic databases of “PsycINFO”, “MedLine (PubMed)”, “Scopus”, “ProQuest Dissertations and Theses”, “CINAHL Plus w/ Full-Text” and “BMC Proceedings”. The above databases were selected to include relevant journal articles, books, and grey literature (e.g., dissertations). Only studies published in English were retrieved as there were no available resources for translation of non-English studies. Studies published between 1994 and 2017 were retrieved; this is because SM was first introduced by Jeffrey Young in 1994. A review of ST for Depression [11] was identified, of which the reference list was examined for cited studies that were eligible for inclusion in the present scoping review. The reference list of all other articles included in the scoping review were not reviewed due to resource limitations. At this stage, a total of 2463 articles were identified for possible inclusion in the scoping review. The processes at the identification stage was conducted by the first author (CL).

#### 2.3.3. Screening Stage

The screening of articles was performed in two stages. The first stage involved screening of titles and abstracts of the 2463 identified articles. At this stage, the screening process was intentionally inclusive and articles were retained if they were relevant to SM and/or ST [13,14]. As “Schema” is a term that has long been used in psychology and not unique to Young, the following checklist was developed to help guide the screening process (see Table 1).

The first author (CL) conducted 100% of the screening at this stage. 10% of the 2463 eligible articles (i.e., 246 articles) were then randomly selected for screening by the second author (JB). In cases where the relevance of an article was not readily apparent, the article was marked for further review by both researchers to determine inclusion in the scoping review. Articles that received a mixed rating were also subjected to a discussion process involving the two researchers before it was determined if the article met the inclusion criteria. Overall, an inter-rater reliability of 95% (calculated using percentage agreement) was observed, indicating that a high level of agreement was observed for decisions on inclusion. At this stage, a total of 1258 articles were identified for possible inclusion in the scoping review. 

#### 2.3.4. Eligibility Stage 

The second screening stage examined titles, abstracts and full-texts for their relevance to SM for depression. The screening process was also deliberately kept inclusive at this stage, where articles were retained if it discussed depression within the context of SM [13,14]. As ST is traditionally used with difficult-to-treat clients, where co-morbidities represent the norm rather than the exceptions, a decision was made to include samples with multiple diagnoses. At this stage, a total of 751 articles were identified for possible inclusion in the scoping review. 100% of the screening at this stage was carried out by the first author (CL) and 10% was carried out by the secondary author (JB). A high inter-rater reliability was observed.

#### 2.3.5. Final Screening Stage

At this stage, only primary research studies testing aspects of the SM for depression were included**.** Articles were included only if depression was the primary diagnosis/focus and measured/tested individually from other psychopathology symptoms. Hence, articles that measured summed psychopathology scores were excluded from the scoping review, e.g., [25,26]. Articles focusing on other disorders (e.g., personality disorders, eating disorders) and depression as a secondary diagnosis were also excluded from the scoping review. Treatment studies, intervention studies, and case studies were also excluded from the scoping review as it does not explicitly test the SM and had also been covered under an existing review [11]. The following checklist helped to guide the selection process (See Table 2).

**Figure 2:**
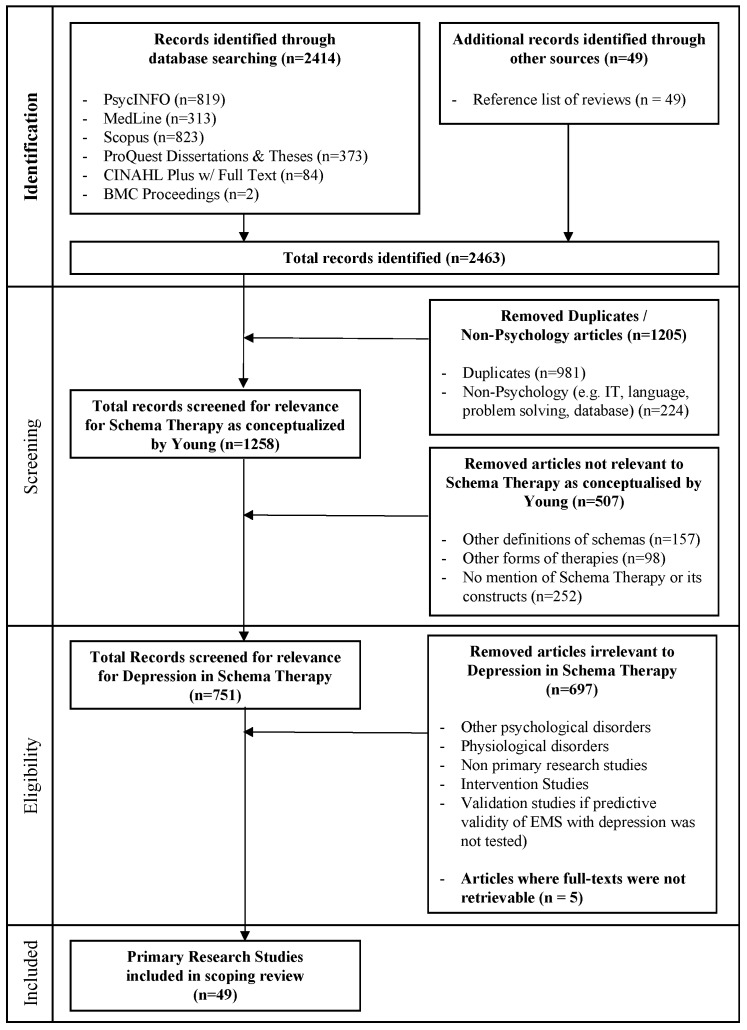
Full screening process in accordance to PRISMA guidelines.

The review decision process at this stage was as follows. The first author (CL) screened the full-texts of all articles at this stage to assess its relevance to depression according to the SM. The second author (JB) simultaneously performed partial screening of titles (10%) to assess its relevance to depression according to the SM. High inter-rater reliability was obtained. A total of 54 articles was identified for inclusion in the scoping review. When full-text articles were not available for download, the researcher contacted authors of these studies to request for its full-text. Five full-texts were not retrievable, leaving the total number of articles included in the final scoping review to be 49. The full screening process is detailed in Figure 2 below.

### 2.4. Data Extraction Process

**Figure 3:**
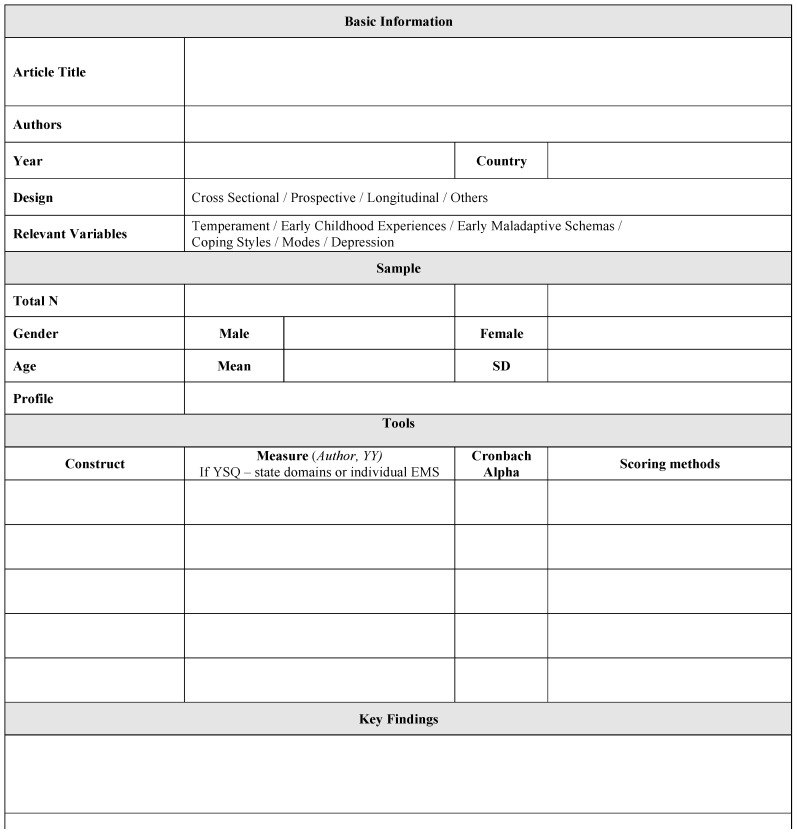
Data extraction form.

The following information was extracted from each of the 49 articles with a data extraction form: (1) basic information including article title, authors, year, country, study design, relevant variables examined in the study, (2) sample characteristics, including total number of participants, gender breakdown, age (mean and standard deviation), and profile, (3) tools, including construct, measure, Cronbach alpha (if reported), scoring methods, (4) key finding including limitations, additional notes, and diagrams of variables tested. See Figure 3 for the data extraction form. The information was then collated into an Excel sheet to obtain the information presented below.

## 3. Results of Scoping Review—Temperament

### 3.1. Overview

Out of the 49 studies included in the scoping review, five examined the relationship between temperament and depression according to SM using scales from two models: The Five Factor Model (FFM) [27] and the Psychobiological Model of Personality (PBM) [28]. See Table 3.

### 3.2. Findings in Relation to ST and Depression

Relevant findings of the five studies are discussed in chronological order, with the discussion focusing on direct and indirect relationships between temperament and depression. Findings relating to other variables (e.g., parental rearing behaviors, early maladaptive schemas (EMS), etc.) as associated or mediating variables will be briefly mentioned but discussed in greater detail in Part II and Part III of the scoping review.

Muris (2006). Maladaptive schemas, perceived parental behaviors, big five personality factors, and psychopathological symptoms in non-clinical adolescents.

[31]

This cross-sectional study was conducted with the objective of assessing perceptions of personality traits, parental rearing behaviors, and psychopathological symptoms among 173 non-clinical adolescents [31]. Muris operationalized temperament with the FFM [27] and measured its five factors as independent variables in the study. Out of the five factors, only neuroticism was found to explain Early Maladaptive Schemas (EMS) and depressive symptoms. Specifically, the relationship between neuroticism and depressive symptoms was mediated by EMS. However, the author acknowledged detrimental parental rearing behaviors as an understudied variable and suggested that future research studies examine broader definitions of parental rearing behaviors.

Halvorsen et al. (2009). Early maladaptive schemas, temperament and character traits in a mixed adult sample.

[34]

This is a cross-sectional study of the relationship between temperament/character, EMS and depression among a sample of clinically depressed, previously depressed and never depressed adult participants [34]. Halvorsen and colleagues operationalized temperament with the PBM [28] and assessed all seven temperament and character traits with the Temperament and Character Inventory (TCI) [28]. High harm avoidance was found to be positively related to 13 EMS and significantly predicted depression severity. Low self-directedness was negatively related to 13 EMS and significantly predicted depression severity. Elevated levels of harm avoidance and self-directedness were also found among the clinically depressed and recovered depressed as compared to never depressed individuals.

Jesinoski (2010) Negative childhood experiences, temperament and negative affectivity among undergraduates.

[33]

This cross-sectional study was conducted among undergraduates to explore direct and indirect relationships among temperament, negative childhood experiences, EMS, and negative affect in adulthood [33]. Temperament was operationalized as neuroticism by the author, justified by its (1) high conceptual similarity to the concept of temperament as proposed by Young; (2) strong empirical basis as one of the most robust components of the FFM of personality; (3) demonstrated relative stability over time; and (4) high correlation with negative affect and depression [31]. While acceptable Cronbach’s alphas were reported for all six neuroticism facets of the Neuroticism Extraversion Openness—Personality Inventory—Revised (NEO-PI-R) [32] (α = 0.60 to 0.84), Jesinoski noted a relatively weaker relationship between the facets of anxiety and depression with the overall neuroticism scale. After considering the conceptual overlap between anxiety and depression subscales and dependent variables of negative affect for the larger theoretical model, he removed the anxiety and depression subscales from the neuroticism scale. He then found strong relationships between neuroticism and 15 EMSs. A consistent and robust effect was also found between temperament and negative affect [33]. Data supported the model as trauma and temperament variables accounted for almost 54% of variance in EMS belonging to Domain 1; Direct and indirect pathways from trauma, temperament and EMS (Domain 1) accounted for 93% of variance in depression. The author found that neuroticism and parental rearing only explained a restricted proportion (maximally 35.7%) of variance in EMS and attributed it to the limited parenting variables examined, and the considerable overlap between the measurement of temperament and adult outcomes of depression.

Balsamo (2013). Personality and depression among undergraduates.

[36]

The fourth study is a cross-sectional study among 230 undergraduates investigating the relationship between personality, anger, and depression [36]. Balsamo operationalised personality with the PBM [28], and assessed all seven measures on the TCI-R. The author found significant correlations between only the character trait of cooperativeness and level of depressive symptoms.

Calvete (2014). Neuroticism, emotional abuse, early maladaptive schemas, and depressive symptoms among adolescents.

[30]

The fifth study is a longitudinal study among 1052 adolescents investigating the relationship between temperament, emotional abuse, EMS and depression/social anxiety [30]. Calvete conceptualized temperament with the FFM, specifically neuroticism and extraversion due to their influence in the development of psychological disorders [30]. Calvete found that neuroticism exerted a direct relationship on depressive symptoms. This study extended existing research with its longitudinal design, where baseline levels of neuroticism and extraversion predicted subsequent depressive symptoms. However, further mediational analyses revealed that only neuroticism at time point 1 significantly predicted increase of depressive symptoms at time point 3 via Disconnection and Rejection EMS (time point 2). The author did not find a significant interaction between temperament and emotional abuse in predicting either EMS or depressive symptoms.

### 3.3. Overall Findings of Existing Research

In conclusion, the following variables were found to have either a direct or an indirect effect on level of depressive symptoms: high neuroticism, high harm avoidance, low self-directedness, and low cooperativeness.

#### 3.3.1. Neuroticism

First, neuroticism was associated with elevated levels of EMS among non-clinical adolescents [30,31] and undergraduates [33]. This was supported both cross-sectionally and longitudinally. Second, neuroticism demonstrated a direct relationship on depressive symptoms among adolescents [30,31] and undergraduates [33]. Third, while the interaction between neuroticism and various types of negative early childhood experiences (ECE) may impact depressive levels, specific relationships are unclear due to the narrow definition of ECE employed by existing studies [31].

#### 3.3.2. Harm Avoidance, Self-Directedness, and Cooperativeness 

Harm avoidance and self-directedness were found to be associated with 13 EMS and depression severity among a mixed sample [34]. The two variables distinguished between the clinically depressed/recovered depressed and never depressed, providing support for their role as predisposing factors in the development of depression. Low cooperativeness was found to be associated with depressive levels among an undergraduate sample [36]. The role of harm avoidance, self-directedness, and cooperativeness in the SM for depression is promising and awaits replication in future research studies.

#### 3.3.3. Ruled out Variables

The following variables were tested but yielded non-significant effects on depressive symptoms: (1) Extraversion (i.e., although found to predict depressive symptoms, this relationship does not appear to be mediated through EMS [33]); (2) Agreeableness, Openness to Experience, Conscientiousness (i.e., these FFM variables did not impact either EMS or depressive symptoms [34,36]); (3) Novelty seeking, Reward Dependence, Persistence, Self-Transcendence (these PBM variables did not impact either EMS or depressive symptoms [34]).

## 4. An Examination of the Two Models

Although temperament variables were found to exert both a direct and an indirect relationship on depression, it is unclear if the Five Factor Model [27] or the Psychobiological Model of Personality [28] is more appropriate to assess the construct of temperament in the SM. Hence, the following section examines both models for its suitability.

Before discussing each model’s compatibility with SM, Young’s conceptualization of temperament in SM will be elaborated upon. According to Young, “emotional temperament” plays a major role in the development of early maladaptive schemas and/or the type of coping styles used [14]. For instance, different temperaments selectively expose children to different life circumstances, and interacts with painful childhood events to form schemas [14]. Two children with different temperaments is also likely to react differently given the same parental treatment. Young and colleagues (2003) observed that an extremely favorable or aversive early environment can override emotional temperament and similarly, an extreme emotional temperament can override an ordinary environment to produce psychopathology [14]. Citing prior research [37], Young and colleagues further postulated temperament to have a biological underpinning (i.e., “largely inborn”) and to be relative stable across lifespan [14].

While Young and colleagues suggested dimensions in conceptualizing emotional temperament, (e.g., labile/nonreactive; dysthymic/optimistic), he highlights that these dimensions are not exhaustive and did not elaborate on ways to measure it [14].

### 4.1. The Five Factor Model (FFM)

The FFM of personality is an empirical model of personality traits [27]. It conceptualizes that personality stems from biological bases such as associated genes and brain structures [38]. The five factors are (1) Neuroticism; (2) Extraversion; (3) Openness to Experience; (4) Agreeableness; and (5) Conscientiousness. The above five factors have been validated in a variety of studies, including peer rating scales [39], self-reports on trait descriptive adjectives [40], questionnaire measures of needs and motives [41], expert ratings on the California Q-Set [42], and among personality disorder symptom clusters [41,43].

Heritability studies have supported the genetic component [43,44] and biological basis [45] of the five factors, and its stability over time [46,47]. This is theoretically compatible with Young’s conceptualization of temperament as being with biological precursors with relative stability over time [14].

Out of the five factors in the FFM, neuroticism appears most closely related to the temperament constructs of inhibition to the unfamiliar [37] and negative affectivity [48] as discussed by Young [14]. Neuroticism represents excessive physiological responsivity of specific brain systems that predisposes individuals to psychopathology [49,50,51]. Within the larger context of research literature non-specific to SM, neuroticism was also found to be associated with many forms of psychopathology and behavioral health, especially depressive disorders [49,52,53].

That said, like any scientific models, the FFM has its limitations. Critics have argued that the FFM does not provide a complete theory of personality [53] as it was developed as a “structural model that provides an account of personality that is primarily descriptive rather than explanatory, emphasizes regularities in behavior rather than inferred dynamic and developmental process” [54] (p. 140). Nevertheless, the FFM provides a conceptual foundation that helps researchers examine theoretical issues surrounding temperament.

### 4.2. Psychobiological Model of Personality (PBM)

The PBM [28] conceptualizes personality as a combination of 4 temperaments and 3 character dimensions. It states that temperament reflects heritable, neuro-biologically based differences, while character refers to differences in self-concepts that develop across the lifespan in response to social-cultural influences. Hence, temperament and character dimensions differ in timeline, rules of operations, brain substrate, and inheritance [28]. The four temperament dimensions are: (1) harm avoidance; (2) novelty seeking; (3) reward dependence; and (4) persistence [28]. For example, harm avoidance refers to an inborn tendency to be pessimistic, fearful, and shy, where individuals high in harm avoidance have high anticipatory anxiety or fear about danger [28]. The three character dimensions are (5) self-directedness; (6) cooperative; and (7) self-transcendence. For example, self-directedness refers to a tendency to be dependent, immature in adapting behavior to define and pursue meaningful goals, where individuals who are low in self-directedness are blaming, undisciplined, and inept.

The Temperament Character Inventory-Revised (TCI-R) [35] is widely used with multiple meta-analyses supporting its strong and consistent predictive validity of depressive symptoms both cross-sectionally and longitudinally [55,56]. Within the context of ST, a few researchers have used TCI to assess temperament [34,36,57]. High correlations between TCI scores and EMS were found among both clinical [34] and non-clinical samples [57], providing support for the validity of part of the SM [14].

However, Farmer and Goldberg found mixed results for TCI’s factorial structure [58]. Citing incongruences with pharmacological and neurochemical research, they concluded that TCI’s psychometric difficulties might be due to inherent issues present within Cloninger’s model [58]. It is also interesting to note that character dimensions were a later addition by Cloninger after discovering that temperament traits alone were not sufficient to distinguish patients from healthy controls [34,57]. 

Further, while some authors [34,57] suggested that the PBM is compatible with temperament as conceptualized in SM, this is not true for all constructs of PBM. For example, temperament traits such as harm avoidance (i.e., a tendency to be anxious, pessimistic, and shy and self-directedness) appears to be theoretically compatible with the SM. Supporting this, studies of mood disorders have found elevated harm avoidance scores among clinical samples as compared to community samples [59,60,61,62]. However, a crucial question about the elevated scores relates to whether they reflect lifelong personality traits or transient mood states: Harm avoidance was found to transiently increased when individuals were agitated or depressed [28,59,61,63], and shown to co-vary with changes in depression [59]. Additionally, character traits (i.e., later developed traits) such as self-directedness (i.e., a tendency to be dependent, immature in adapting behavior to define and pursue meaningful goals) and cooperativeness are theoretically incompatible with Young’s conceptualization of temperament as being biological and heritable.

### 4.3. Overall Suitabaility to Assess Temperament

Hence, between the two models, the FFM is deemed as being more suitable to operationalize temperament in studies of SM and depression due to the following reasons: (1) its theoretical compatibility with SM; (2) established psychometric properties for its factors and measures (e.g., the NEO-PI-R); and (3) its widespread use both among studies of, and external to SM. 

## 5. Limitations

The above findings should be considered in context of several limitations. First, the relationship between temperament and depression was mostly examined among non-clinical samples, potentially limiting its generalizability to clinical samples. Second, the majority of studies in this review had sampled adolescents and undergraduates, again limiting the generalizability of its findings to adult samples. Third, only primary studies published in English was included, resulting in a small number of studies for inclusion in this review. Future research studies can consider studying the relationships between temperament and depression according to the SM among adult clinical samples.

## 6. Conclusions

This scoping review examined effects of temperament on depression in context of the SM. Five studies were identified, where researchers operationalized temperament using measures from either the Five Factor Model (FFM) or the Psychobiological Model of Personality (PBM). Overall, studies found that variables of high neuroticism, high harm avoidance, low self-directedness, and low cooperativeness exerted a direct and/or an indirect effect on level of depressive symptoms. As it was unclear which of the two models was more appropriate to assess the construct of temperament in SM for depression, both models were examined for their suitability.

The FFM has several strengths. First, it is theoretically compatible with SM as both conceptualized temperament as being with biological precursors and relative stability over time. Second, the psychometric properties of its factors and measures are well-established. Third, the link between neuroticism and psychopathology was supported and replicated by studies both within and external to SM. Limitations of the FFM included its primarily descriptive nature and lack of emphasis on individual dynamic and developmental process. Strengths of the PBM lies in the strong psychometric properties of its measure and its use among adult clinical samples by a SM study. However, there are concerns with the PBM’s factorial structure, its ability to discriminate between patients and controls, and its incongruences with pharmacological and neurochemical research relating to its biological basis. In addition, three out of seven traits in the PBM are character traits. Character traits develop across the lifespan and are theoretically incompatible with Young’s biological conceptualization of temperament

In conclusion, the FFM is deemed as more suited to operationalize temperament in studies of SM and depression due to its theoretical compatibility with SM, established psychometric properties of its factors and measures, and widespread application both within and external of studies of SM. Out of the five factors in the FFM, only neuroticism was found to exert direct and indirect effects on depression. Despite the small number of studies reviewed and homogenous sampling, this review represents a first step in the systematic examination of the empirical basis of SM and a contribution to treatment innovation and practice for depression.

## Figures and Tables

**Table 1:** Checklist to guide first stage screening for scoping review.

Are Articles Relevant to SM [13,14]?	
Articles Containing the Following Terms Are	
Likely to Be Relevant	Unlikely to Be Relevant
- Schema Therapy - Dual Focused Schema Therapy - Schema Focused Therapy - Schema Therapy Model - Schema Model - Early Maladaptive Schemas - Young’s Schema Questionnaire (YSQ) - Early Maladaptive Schema Questionnaire (EMSQ) - Modes - Schema Mode Inventory (SMI)	(Other Definitions of Schemas) - Knox’s Image Schema Model - Self Schema Model (of Emotions) - Schematic Processing Model - Emotional Schema Model/Therapy - Leahy Emotional Schema Scale - Family Relational Schema - Victim Schema Model - Beck’s Schema Model - Integrative Self-Scheme Model - Markus’s Schema Model - Relational Self-Schema Model - Moral Schemas - Spiritual Self-Schema Therapy - Schematic Influences - Body Schemas
	(Other Therapies) - Dialectical Behavioural Therapy (DBT) - Functional Analytic Psychotherapy (FAP) - Cognitive Behavioural Therapy (CBT) - Rogerian Supportive Therapy - Compassion Focused Therapy
	(Others) - Imagery re-scripting as a stand-along technique - Use of schema tools but in cognitive paradigms instead of psychotherapy context - Introduction to special issues
	(If there were no mention of Schema Therapy and its related constructs in article’s title and/or abstract)

**Table 2:** Checklist to guide final stage screening for scoping review.

Are Articles Relevant to Pathways to Depression within ST [13,14]?	
Articles Containing the Following Terms Are	
Likely to Be Relevant	Unlikely to Be Relevant
- Depression/Depressive/Depressed - Major Depressive Disorder - Dysphoria - Mood Disorders - Validation studies (explicit testing of predictive validity with depression)	- Personality Disorder - Sexual Dysfunction - Psychosis - Post-Traumatic Stress Disorder - Bipolar Disorder - Obesity - Eating Disorder - Obsessive Compulsive Disorder - Substance Dependence - Work Dysfunction - Anxiety Disorder/Social Anxiety Disorder - Validation studies (did not explicitly test predictive validity with depression) - Treatment Studies - Randomized Controlled Trials - Interventions - Single Case Studies - Schema questionnaires that were not used to measure constructs - Non-primary research studies (opinions, books) - Antidepressant treatments on EMS

**Table 3:** Summary of studies in scoping review examining temperament.

Construct	Model	Tool/Author/Year	Studies
Temperament	Five Factor Model	Big Five Questionnaire—Children (BFQ-C) [29]	Calvete, 2014 [30]; Muris, 2006 [31]
	Five Factor Model	Neuroticism Extraversion Openness—Personality Inventory—Revised (NEO-PI-R) [32]	Jesinoski, 2010 [33]
Temperament & Character	Psychobiological Model of Personality	Temperament & Character Inventory (TCI) [28]	Halvorsen et al., 2009 [34]
	Psychobiological Model of Personality	Temperament & Character Inventory—Revised (TCI-R) [35]	Balsamo, 2013 [36]
